# Gold Nanostars with Reduced Fouling Facilitate Small Molecule Detection in the Presence of Protein

**DOI:** 10.3390/nano11102565

**Published:** 2021-09-29

**Authors:** Anastasiia Tukova, Inga Christine Kuschnerus, Alfonso Garcia-Bennett, Yuling Wang, Alison Rodger

**Affiliations:** 1 Department of Molecular Sciences, Faculty of Science and Engineering, Macquarie University, Sydney, NSW 2019, Australia; alf.garcia@mq.edu.au (A.G.-B.); alison.rodger@mq.edu.au (A.R.); 2 Electron Microscopy Unit, University of New South Wales, Sydney, NSW 2052, Australia; i.kuschnerus@unsw.edu.au; 3 School of Materials Science and Engineering, University of New South Wales, Sydney, NSW 2052, Australia

**Keywords:** gold nanoparticles, nanostars, nanospheroids, protein corona, surface-enhanced Raman spectroscopy, circular dichroism, cryo-TEM

## Abstract

Gold nanoparticles have the potential to be used in biomedical applications from diagnostics to drug delivery. However, interactions of gold nanoparticles with different biomolecules in the cellular environment result in the formation of a “protein corona”—a layer of protein formed around a nanoparticle, which induces changes in the properties of nanoparticles. In this work we developed methods to reproducibly synthesize spheroidal and star-shaped gold nanoparticles, and carried out a physico-chemical characterization of synthesized anionic gold nanospheroids and gold nanostars through transmission electron microscopy (TEM), dynamic light scattering (DLS), zeta potential (ZP), nanoparticles tracking analysis (NTA), ultraviolet-visible (UV–Vis) spectroscopy and estimates of surface-enhanced Raman spectroscopy (SERS) signal enhancement ability. We analyzed how they interact with proteins after pre-incubation with bovine serum albumin (BSA) via UV–Vis, DLS, ZP, NTA, SERS, cryogenic TEM (cryo-TEM) and circular dichroism (CD) spectroscopy. The tests demonstrated that the protein adsorption on the particles’ surfaces was different for spheroidal and star shaped particles. In our experiments, star shaped particles limited the protein corona formation at SERS “hot spots”. This benefits the small-molecule sensing of nanostars in biological media. This work adds more understanding about protein corona formation on gold nanoparticles of different shapes in biological media, and therefore guides design of particles for studies in vitro and in vivo.

## 1. Introduction

Gold nanoparticles (GNPs, used to denote any size or shape nanoparticles) are among the most widely studied nanomaterials. Since the primary report on gold colloids over 100 years ago by Faraday [1,2], extensive research has been performed on their configurations, properties, and applications. GNPs exhibit a myriad of unique physical, optical, biological and chemical features [3,4,5,6]. Recently, the utilization of GNPs in disease treatment has been examined because research has indicated the capacity of GNPs to target and kill microscopic organisms and tumor cells [7,8,9,10,11].

When incident light is applied to metal nanoparticles, due to the comparable size of the particles and the wavelength of the incident light, the effect of localized surface plasmon resonance (LSPR) occurs [12]. Electromagnetic fields close to GNP surfaces can be amplified up to several orders of magnitude by this effect. The biggest enhancements are localized in so-called “hot spots”, which have been found to occur in the areas of the highest local curvature or in the gaps between two GNPs. An LSPR “hot spot” on a particle enhances the electromagnetic field for neighboring molecules. This has been useful in surface-enhanced Raman spectroscopy (SERS) and surface-enhanced fluorescence (SEF) [6].

Anisotropic plasmonic nanoparticles have been found to provide additional Raman signal enhancement for analytes located close to tips and corners which form “hot spots” [13,14,15,16]. Gold nanostars (AuNS) with multiple “spike”-like branches have many “hot spots” per particle and have recently garnered significant attention because of their ability to amplify and absorb light in the NIR and the visible regions [17,18,19,20,21], which enables various applications that require deep tissue penetration, such as bio-imaging and photo-thermal cancer treatment [13,22]. AuNS demonstrate higher enhancement factors than other shapes at their resonance wavelengths, enabling zeptomol detection [14,23,24,25].

Although GNP designs and functional uses are rapidly developing, little is known about the changes that nanoparticles undergo and how they behave in complex biological systems. We know that interactions with different proteins in the cellular environment result in the formation of the “protein corona” (PC)—a layer of proteins formed around a nanoparticle. A PC can be characterized as “hard” or “soft”, depending on the strength of protein adhesion (often dependent on the distance from the surface) to the nanoparticle’s surface [26,27]. The PC formation on the GNPs induces variations in the SERS enhancement of the particles due to aggregation and morphology changes and simply precluding analyte binding to “hot spots”. The PC also influences the targeting efficiency of the nanoparticles [28]. Literature reports suggest that less than 5% of the GNPs injected into a biological system reach targeted organs [29,30], due to aggregation, shape altering and surface chemistry changes to the particles and to the natural bio-distribution of GNPs into each organ [31,32]. Considering possible particle accumulation, toxicity also becomes important [33,34,35,36,37].

Protein binding to the GNP surface is a complex process that is affected by the structural properties of the protein [36,38,39,40], and the size, shape and surface chemistry of the GNPs [41,42]. It may result in conformational changes of the protein. As the PC becomes the outer surface of the GNPs, it modulates the bio-identity of GNPs, and hence, their overall pharmacological and toxicological profiles. The investigation of protein interactions with GNPs is therefore an important step towards any biomedical application.

In this study, we first report the reproducible synthesis of anionic, citrate-capped spheroidal and ascorbate-capped star-shaped GNPs, which was required in order to be able to undertake a reliable protein corona study. We report their structured physico-chemical characterization, including the shape, size, optical properties and Raman signal enhancement ability. We also analyze how the two differently shaped GNPs interact with proteins following pre-incubation with bovine serum albumin (BSA), the most abundant protein in blood plasma (50–60% of plasma proteins). We demonstrate GNP–protein interactions using UV–visible (UV–Vis) spectroscopy, dynamic light scattering (DLS), zeta potential (ZP), nanoparticle tracking analysis (NTA), cryo-TEM and surface-enhanced Raman spectroscopy (SERS). Protein conformational changes were examined via circular dichroism (CD) spectroscopy. The results demonstrated limited PC formation on the star-shaped particles, which could benefit their performance in biological media. BSA underwent sleigh conformational changes upon adsorption on the nanospheroid surface with some unfolding of α-helical structure, which presumably affects the biological function of BSA. We also analyzed how gold nanospheroids and nanostars interact with small molecules after pre-incubation with protein. Gold nanostars demonstrated reduced fouling, which significantly improved the SERS signal magnitude of small molecules in the presence of BSA compared to gold nanospheroids.

## 2. Materials and Methods

### 2.1. Reagents

Reagents for the synthesis of GNPs and other tests were purchased from Sigma-Aldrich (Sydney, Australia) unless stated otherwise. For all aqueous solutions, MilliQ water (18.2 MΩ· cm (at 25 °C) was used.

### 2.2. Preparation of Nanoparticles

Spheroidal GNPs (following convention, we use AuNP exclusively for spheroidal particles) were prepared through an adjusted with Turkevich’s method [43,44], using tetrachloroauric acid (HAuCl_4_) as the precursor and sodium citrate (Na_3_C_6_H_5_O_7_) as the reducing agent. First, 0.01% (*w*/*v*) tetrachloroauric acid (aqueous solution) in a 100 mL glass flask was stirred with the bar at a constant speed using a magnetic mixer set at 550 rpm and heated until it boiled, which was followed by the addition of a 1% (*w*/*v*) trisodium citrate aqueous solution with a 1:2 volume ratio (Na_3_C_6_H_5_O_7_:HAuCl_4_). After approximately 5 min, a progressive change of the solution’s color was apparent. The solution was then left to boil with continuous stirring for 20 min. The transparent solution first became dark colored (grayish-blue), indicating the formation of gold seeds, which then changed color to ruby as the particles’ concentration and size changed. This color change indicated the formation of GNPs. Heating was turned off after 20 min and the mixture was stirred for further 10 min. When the mixture had cooled to room temperature, it was stored below 4 °C until further use.

AuNS were produced through a seedless, surfactant-free synthesis in aqueous solution at ambient temperature. In a variation on the AuNP synthesis, tetrachloroauric acid was used as the precursor, while ascorbic acid (C_6_H_8_O_6_) acted as the reducing agent and silver nitrate (AgNO_3_) as the shaping agent in the preparation of AuNS [45]. In a glass beaker, 10 mM AgNO_3_ was mixed with 10 mM HAuCl_4_ in 1:18 volume ratio for 30 s. The solution color was slightly yellow, due to the presence of yellow gold salt ions. After that, we slowly (drop-wise) added (C_6_H_8_O_6_) (100 mM) with a 1:6 *v*/*v* AgNO_3_:C_6_H_8_O_6_ ratio. The mixture was then stirred for another 30 sec until the color of the solution turned blue. The synthesized particles were stored in the refrigerator below 4 °C until further use.

To prepare GNPs with a PC, GNPs were incubated in a 4.5 mg/mL BSA (Sigma, Sydney, Australia, A9647, heat shock fraction) solution for 30 min at room temperature and under ambient light. The AuNP:BSA mass ratio values were approximately 1:90 and 1:64 for AuNP and AuNS, respectively. After incubation, samples were centrifuged at 10,000 rpm at 4 °C for 5 min. The supernatant (containing free protein) was carefully removed and discarded. The pellets were suspended in water and briefly sonicated for a few seconds. This was repeated twice. The three-step suspend, sonicate and centrifuge was counted as one wash. We performed one, two or three washing cycles to estimate the effects of multiple purification steps on PC layers on the surfaces of the particles. Prepared samples were stored at −20 °C.

### 2.3. Characterization of Nanoparticles

GNP concentrations were obtained with nanoparticle tracking analysis (NTA) on NanoSight NS300 instrument (Malvern Panalytica, Malvern, UK). Each GNP sample was diluted 10 times from original concentration and run through detector at 50 units syringe pump speed, at 21 °C. Particles were irradiated with a 405 nm laser (see ESI Appendix A.1).

#### 2.3.1. Size and Morphology Analysis

Transmission electron microscopy (Philips CM10 TEM, Eindhoven, The Netherlands) was used to estimate the size and morphology of nanoparticles. All images were taken at a high voltage of 100 kV and magnification of ×92,000. The samples for TEM were prepared by adding a 10 µL drop of an aqueous nanoparticle suspension on top of the carbon-coated copper grid (Zhongjingkeyi Film Technology, Beijing, China), and letting it settle for 3 min before removing the drop with the filter-paper. This was then repeated three times. Then the sample was left to dry for at least 12 h. Particle size distribution analysis from TEM images was performed via *ImageJ*. The instructions for particles size analysis can be found on the application’s web site: https://imagej.net/ImageJ (accessed on 28 September 2020) [46]. Formulas for surface area calculations are in ESI Appendix A.2.

Dynamic light scattering (DLS) measurements were performed to determine particle-size data using the Zetasizer ZS instrument (Malvern Panalytica, Malvern, UK) set at 25 °C. Particles were irradiated with a 633 nm laser with 4 mW power output. Samples were placed in the Zetasizer ZS in disposable folded capillary cells DTS1070 (Malvern Panalytica, UK). To investigate the surface charge, ZP measurements were performed on the same instrument. The samples were diluted from the original concentration with water in a 1:1 ratio. Each sample was measured 3 times over 11 runs.

#### 2.3.2. Study of Optical Properties

The UV–Vis spectroscopy analysis of the nanoparticles’ optical properties was performed on a JASCO V-760 UV–Vis Spectrometer: 400 µL of a sample was placed in a Starna 18B/Q/10 cuvette and scanned from 300 to 900 nm.

For the surface-enhanced Raman spectroscopy enhancement analysis, we used 2,3,5,6-tetrafluoro-4-mercaptobenzoic acid (TFMBA) and 4-mercaptobenzoic acid (MBA) as molecules that give distinctive Raman peaks. First, 10 µL of 1 mM TFMBA/ethanol or MBA/ethanol solution was mixed with 60 µL of each type of nanoparticle. The samples were incubated overnight at room temperature, in the dark, to form a completely self-assembled mono-layer of TFMBA (MBA) molecules on the GNPs’ surfaces; and then centrifuged at 7000 rpm for 10 min, decanted and dispersed in water, thereby removing ethanol and unbounded TFMBA (MBA) molecules. Raman spectra were collected with a portable IM-52 Raman microscope (Snowy Range Instruments, USA), 70 mW of 785 nm incident laser power with an integration time of 60 ms. We recorded and averaged 40 measurements for each spectrum [45]. The spectra were baseline-corrected using the *Peak* software (Snowy Range Instruments, Laramie, WY, USA). The SERS enhancement factor (EF) of GNPs was calculated as outlined in ESI Appendix A.2.

The same measurement parameters were used to collect protein-coated particles’ characterization data.

### 2.4. Protein Corona Characterization

#### 2.4.1. Cryogenic Transmission Electron Microscopy (Cryo-Tem)

Approximately 1 mL (0.06 mg/mL of particles) of each GNP/protein sample (GNP incubated with 4.5 mg/mL BSA and washed one, two or three times) was prepared as a stock solution for cryo-TEM and 4 µL applied as a droplet onto a glow-discharged grid (R2/2 Quantifoil copper grids, Wetzlar, Germany) and blotted with filter paper for 2 s. The grid was plunged into liquid ethane with a Leica EM GP freeze plunger (Leica, Germany) at 10 °C, 89% humidity and stored in liquid nitrogen. Images were acquired using a Talos Artica TEM (Thermo Fisher Scientific, Waltham, MA, USA) with an acceleration voltage of 200 keV.

#### 2.4.2. Protein Secondary Structure Analysis

In order to characterize and estimate the secondary structure of the protein adsorbed on the surfaces of GNPs, we collected circular dichroism (CD) spectra of the protein-coated samples using a Jasco J-1500 CD spectrometer (Jasco, Hachioji, Japan). First, 150 µL of sample was placed in a 1 mm path length quartz cuvette and data were collected from 300 to 190 nm at a scan rate of 100 nm/min. Each spectrum is an average of 3 scans. Background measurements of water were recorded with the same settings and subtracted from the samples’ spectra. The secondary structure was analyzed via a self-organizing map (SOM) structure-fitting methodology (SOMspec) [47]. SOMspec requires a reference set of spectra for proteins of known structure and produces output that enables the user to interrogate what is behind secondary structure estimates.

### 2.5. GNP Interaction with Small Molecules after Incubation with Protein

In order to investigate GNPs’ ability to enhance Raman signals in biological media, we performed the following test. Protein coated GNPs were prepared as previously described. For this test we used samples incubated with BSA and washed once. Then we added 167 µL of 1 mM TFMBA/ethanol or 1 mM MBA/ethanol to 1 mL of protein-coated GNPs aqueous solution and incubated the mixture for 1 h under ambient light. The samples were centrifuged and resuspended in water to remove unbound molecules. The Raman spectroscopy measurements were performed as described above.

## 3. Results and Discussion

### 3.1. Synthesis and Nanoparticles Characterization

#### 3.1.1. Nanoparticles Morphology

**Figure 1:**
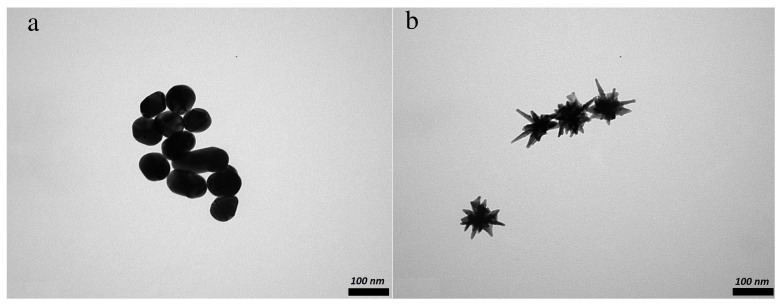
TEM images of GNPs: (**a**) spheroidal GNPs (AuNP); (**b**) star-shaped GNPs (AuNS).

Anionic AuNP, prepared through a modified Turkevich’s method involving reduction of chloroauric acid with sodium citrate in boiling water, have smooth, rounded surfaces and an elongated spheroidal shape. With the synthetic procedure described in the Materials and Methods section, particles of similar size and morphology, slightly elongated spheroids with a diameter of 94 nm, were produced (Figure 1a, Table 1). Reproducibility was confirmed by DLS measurements of different batches (see ESI Appendix B.1). It should be noted that the aggregation state of the particles presented in the images (Figure 1) does not reflect their state when suspended in the solution, as measured by UV–Vis spectroscopy and DLS. The apparent aggregation in the images occurred during drying of the sample.

By way of contrast, reproducible preparation of AuNS was challenging. The particles’ shapes and sizes varied from batch to batch and within batches. Repeat preparations proved to be very sensitive to the implementation of the mixing regime; the type and strength of mixing influenced the particles’ shape. The same issue has been described previously [13,48]. We therefore tried to vary the mixing mode systematically whilst keeping the same chemical ratio, injection time and order of reagent addition (ESI Appendix B.2). Consistent morphology was achieved by mixing reagents with a magnetic stirrer (1000 rpm) with stops for each reagent injection. Visually, the particles from this approach appeared monodispersed and of similar shape. The branches were long and sharp, which was the targeted morphology (Figure 1b) to optimize hot spots. The size of the anisotropic particles prepared by this method was also close to the size of spheroidal particles, which facilitated comparison of their practical application as SERS enhancers. The DLS data analysis shows the size distribution of the star-shaped particles to have been around 139 ± 27 nm. TEM data provided us with morphological details, such as core size (around 50 nm) and branch length (around 46 nm).

The surface charge (zeta potential, ZP) of the AuNP and AuNS was approximately the same due to similar effective surface areas of AuNP and AuNS and similar capping agent distribution on their surfaces (Table 1). The ZP of the prepared particles was in acceptable range to resist aggregation (ZP < −25 mV and >+25 mV) [49,50].

#### 3.1.2. Optical Properties of Nanoparticles

**Figure 2:**
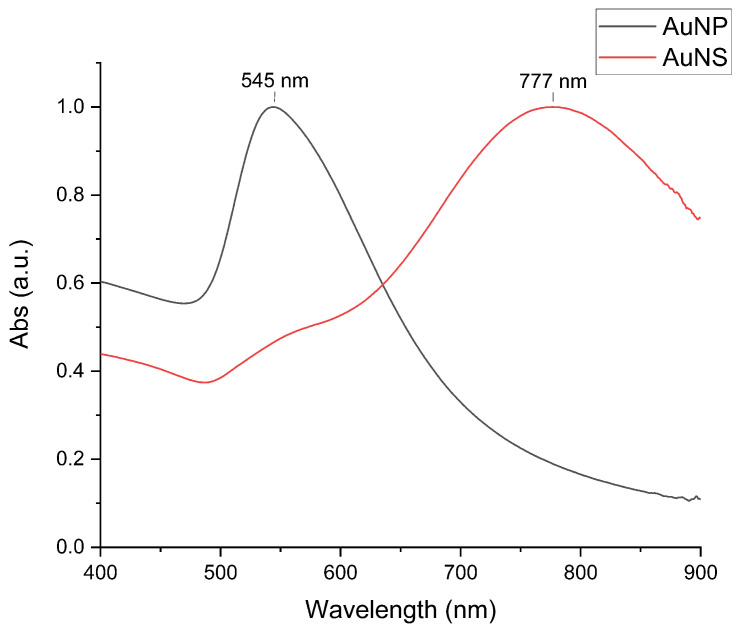
Normalized UV–Vis spectra demonstrating the LLSPR peak position of spheroidal particles (AuNP) at 545 nm and star-shaped particles (AuNS) at 777 nm.

The UV–Vis spectrum of each sample is shown in Figure 2. AuNP have a single LLSPR peak at around 545 nm, whereas AuNS have a small shoulder at around 545 nm and a more intense red-shifted peak at 777 nm. The AuNS are anisotropic particles whose less intense peak at approximately 545 nm is attributable to the LLSPR along the transverse direction (central body (core)), similar to that of AuNP. The dominant second peak at 777 nm arose from the surface plasmon in the longitudinal direction which is attributed to the hybridization of plasmon modes of the body and the individual tips [25]. Since the peak positions in the UV–Vis spectra of the AuNP and AuNS are functions of particle size and shape [12], the peak position of a GNP preparation provided a quick indication of whether we have reached the desired particle size and morphology. As a general rule [25], the further the peak is into the NIR-region, the sharper and longer the nanostars’ branches. The width of the absorption curve indicates the size distribution of the particles: a wide band means high polydispersity and a narrow band is an attribute of monodisperse particles. Our UV–Vis spectral band widths correlate with the PDI (polydispersity index) values in Table 1, with AuNP particles having a narrower size distribution than AuNS. This fact is supported by DLS and TEM data, which show that the AuNS particles are more polydisperse than AuNP (Figure 1).

#### 3.1.3. Raman Signal Enhancement Ability of Nanoparticles

**Figure 3:**
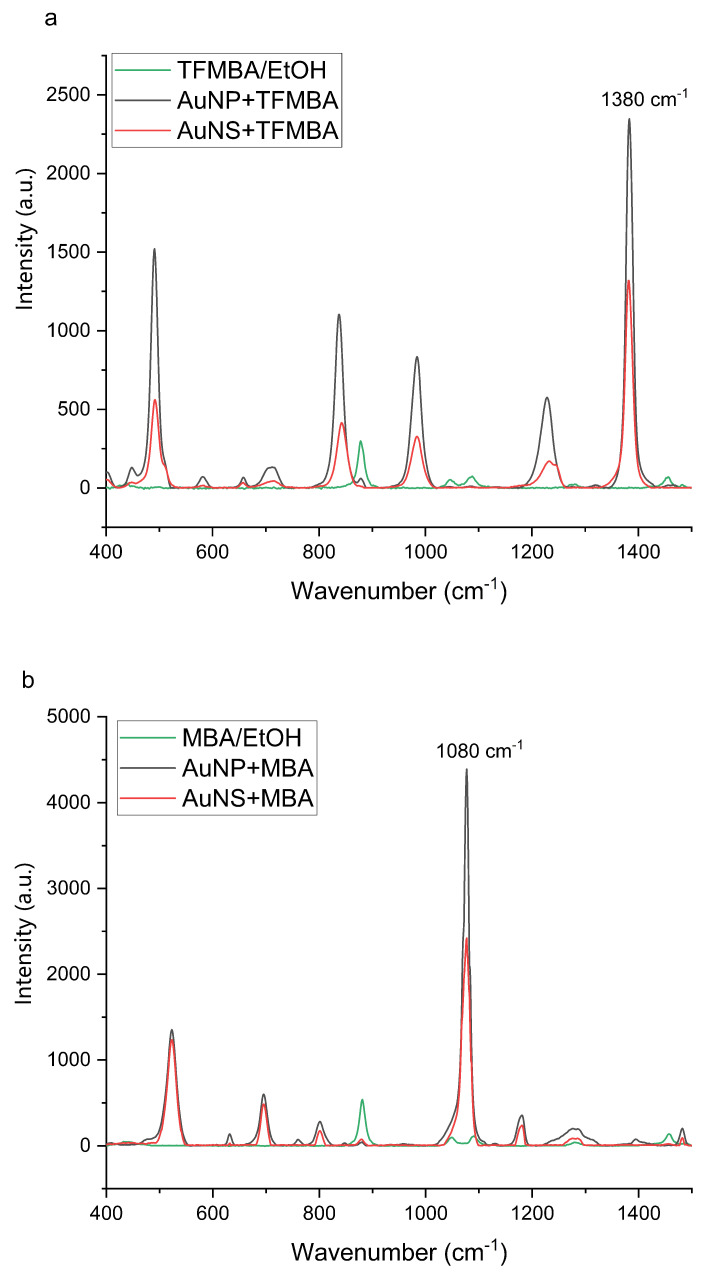
SERS spectra of GNP with (**a**) TFMBA or (**b**) MBA: 60 µL of GNP incubated with 10 µL of 1 mM TFMBA/MBA, followed by removing of the ethanol and free TFMBA and the particles resuspended in water (black line $1.8\times 10^{10}$ particles/mL AuNP, pink line $2.9\times 10^{9}$ particles/mL AuNS). A spectrum of 1 mM TFMBA (MBA) in ethanol is overlaid.

Next, we investigated the ability of GNPs to enhance Raman signals *via* SERS analysis with the TFMBA and MBA. In Figure 3, the green line of TFMBA Raman spectrum is for 60 µL of 1 mM TFMBA/MBA in ethanol. The concentration of analytes in ethanol was insufficient to have a distinctive Raman; the observed peaks of the green line were only from ethanol (TFMBA and MBA Raman spectra without solvent can be viewed in ESI Appendix A.3). The Raman spectra of the analytes in the presence of both GNPs, as illustrated in Figure 3, demonstrated enhanced signals for the distinct peaks of TFMBA at 1380 ${\mathrm{cm}}^{-1}$ and MBA at 1080 ${\mathrm{cm}}^{-1}$; the two particle types gave similar enhancement factors (EF) when adjusted for concentration of particles as summarized in Table 2. Ethanol is not apparent in the GNP–analyte spectra (the enhanced SERS spectra) (black and pink lines), as during the sample preparation, the particles, conjugated with TFMBA/MBA, were washed, centrifuged and resuspended in water, so ethanol was removed and only peaks from TFMBA/MBA on the surfaces of GNP were observed. The Raman signal enhancement (SERS) in the presence of the GNPs is attributed to the localized plasmon resonance effect of metal nanoparticles with the TFMBA/MBA bound close to the surface (approximately 1 µM of TFMBA/MBA in each sample; see ESI Appendix A.2). The difference in signal magnitude of AuNP and AuNS was observed over multiple tests and was due to the different particle concentrations in the samples and the difference in the particles’ ability to create hot spots (these differences are considered in EF calculations summarized in Table 2 (ESI Appendix A.2).

#### 3.1.4. Changes of Nanoparticles Properties after Interaction with Protein

**Figure 4:**
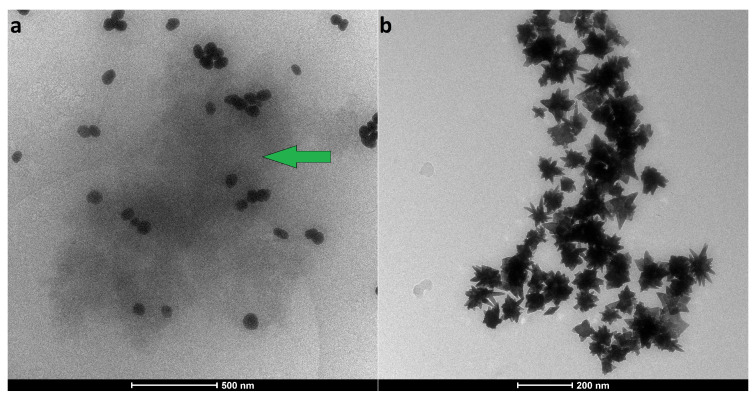
Cryo-TEM images of protein-coated GNPs: (**a**) AuNP+BSA (after first wash); (**b**) AuNS+BSA (after first wash). Soft PC is indicated with the green arrow.

Cryo-TEM was used to visualize the GNPs after incubation with BSA (Figure 4). Both spheroidal and star-shaped nanoparticles could be seen as intact regarding their original morphology and size. Incubation with protein did not cause any morphological changes, although the AuNP were surrounded with a protein cloud (“soft” corona [26]) (as seen in Figure 4a). The observed contrast which we attribute to the protein is consistent with our previous TEM work on the protein corona of gold [51]. Images of AuNS samples incubated with BSA do not have visible signs of protein around the particles (Figure 4b), even at higher magnifications (see ESI Appendix D). Despite the fast freezing cryo-TEM sample preparation method, the cryo-TEM images do not necessarily represent the solution-state aggregation of particles, as discussed below.

**Figure 5:**
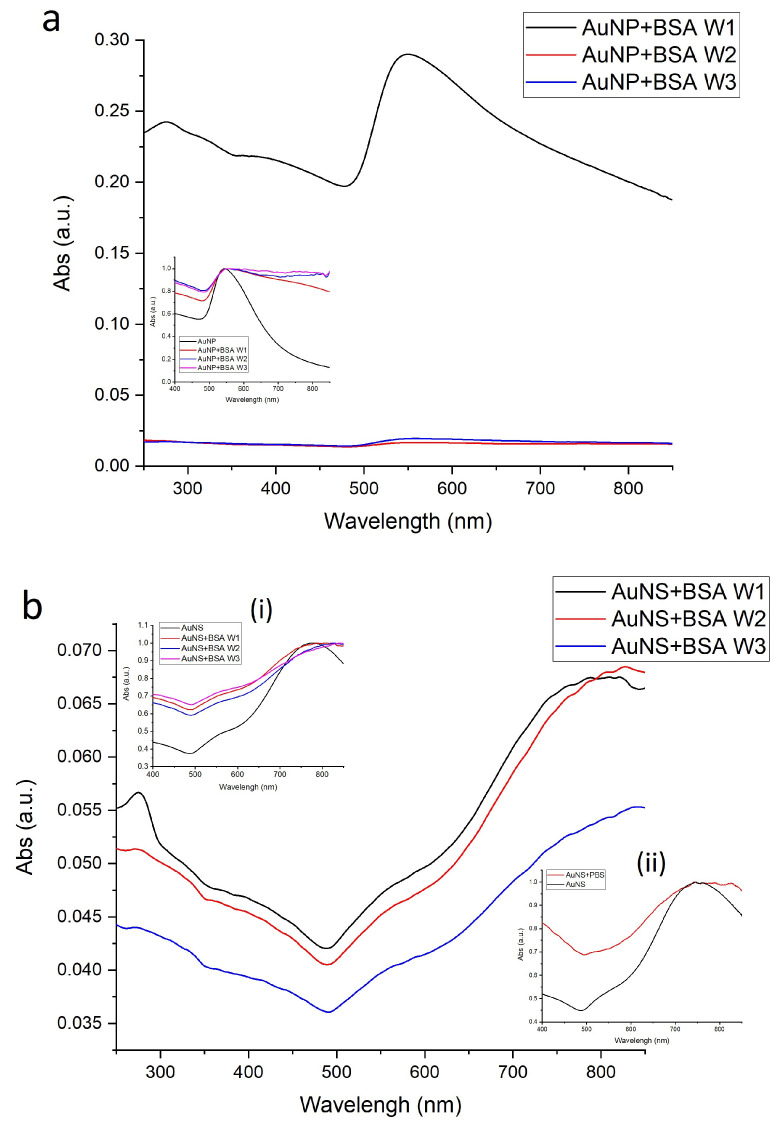
UV–Vis absorbance of protein-coated GNPs after first (W1), second (W2) and third (W3) wash cycles. (**a**) AuNP—spheroidal particles, inset: normalized spectrum; (**b**) AuNS—star-shaped particles, insets: (i) normalized spectrum and (ii) normalized UV–Vis spectrum of AuNS aggregation in PBS.

**Figure 6:**
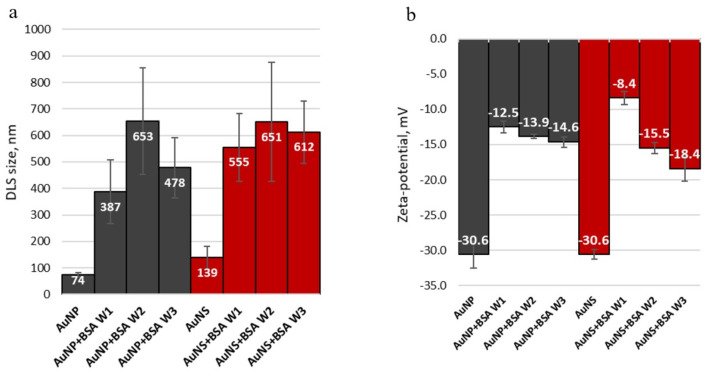
Size and charge of the protein-coated particles after first (W1), second (W2) and third (W3) wash cycles: (**a**) DLS size data for GNPs and protein-coated GNPs, and (**b**) ZP surface charge data of GNPs and protein-coated GNPs.

Incubation of GNPs with BSA resulted in changes in LSPR, as demonstrated by changes in the absorption spectra, such as a red-shift of the LSPR position by up to 10 nm, peak broadening and decreased signal intensity compared to bare GNPs of Figure 5. Although the red-shift can indicate that a dense dielectric layer of protein has formed on the surface of the nanoparticles [52,53], it can also indicate aggregation of the particles. When GNPs aggregate, they behave as larger particles with a LSPR absorbance maximum at a longer wavelength (red-shift) [54]. For the AuNP samples, after the first wash (Figure 5a) which removed only the unbound protein, a thick “soft” PC was formed, causing the particle size (Figure 6a) to increase relative to the protein-free samples. After the second and third washes, as more protein was removed (see ESI Appendix D), the particles aggregated (we speculate there was no capping agent to keep the particles dispersed), causing a further increase in size. In the case of the AuNS, there were no visible protein layers formed around the particles, so their size changes (Figure 6a) were most likely cased by the particle aggregation after capping agent removal with the washing cycles. The DLS data correlate with this observation (Figure 6a).

**Figure 7:**
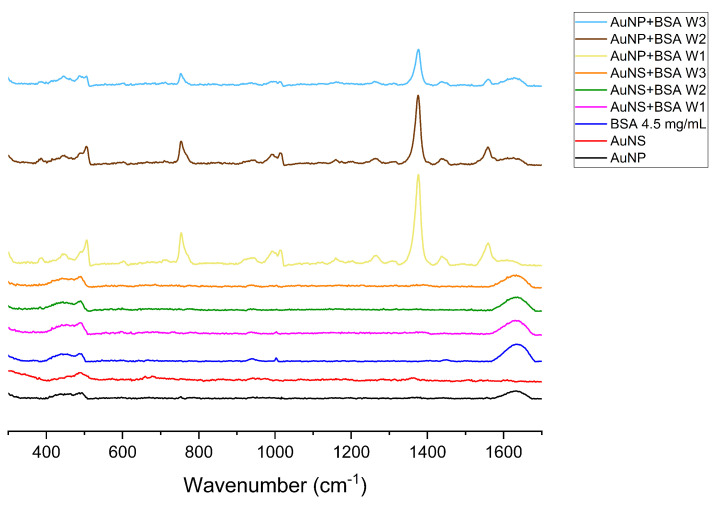
SERS spectra of protein plus particles after first (W1), second (W2) and third (W3) wash cycles: AuNP (spheroidal GNPs), AuNS (star-shaped GNPs). For the Raman signal of protein without GNPs we used 60 µL of 4.5 mg/mL BSA solution.

A change in surface-charge of both GNP was detected after incubation with the protein (Figure 6b). The decrease in the ZP of AuNP was caused by the protein-coat screening the particles’ negatively charged surfaces due to the positively charged BSA residues, such as arginine, histidine and lysine, electrostatically interacting with the negatively charged surfaces of the GNPs particles [55] tending towards the mean ZP of a BSA solution, of −14.95 ± 9.4 mV at pH = 5.6 [56]. As layers of protein were removed by successive washes (protein signal intensity decreases in Raman spectra Figure 7), the surface charge gradually became more negative. The AuNS charge reduced even more than the AuNP, which we attribute to the removal of capping agent with the washing cycles results, concomitant with the sharp increase in size of the particles with the aggregation.

### 3.2. Protein Corona Characterization

#### 3.2.1. Analysis of the Protein–Particle Interactions

SERS spectra for GNPs, BSA and GNPs plus BSA are shown in Figure 7. The bands observed and their assignments are summarized in Table 3.

Overall, Raman vibrational peaks of AuNP+BSA samples have significantly higher intensity than AuNS+BSA (Figure 7), which suggests little or no binding of BSA to the surfaces of the particles.

Some of the well-known characteristic peaks of BSA, such as the Tyr doublet (850–830 ${\mathrm{cm}}^{-1}$), are not included in Table 3, as it is not present above the noise level in our spectrum [57,58]. There is a relationship between the intensity of the peaks of certain residues and distance from the GNP surface. Due to the structure of BSA, tyrosines and tryptophans were buried in the hydrophobic core so that their SERS enhancement was smaller than other transitions, as they would not be at the GNP surface [59].

#### 3.2.2. Conformational Changes of Protein

**Figure 8:**
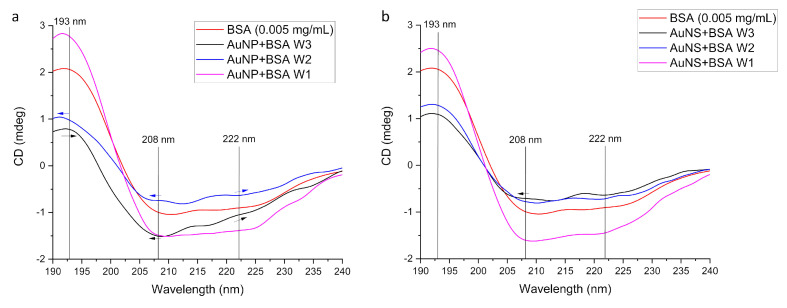
CD spectra of protein-coated GNPs: (**a**) AuNP, (**b**) AuNS. For the CD analysis we used 150 µL GNP incubated with BSA (4.5 mg/mL) and washed one (W1), two (W2) or three (W3) times and 150 µL of BSA solution (0.005 mg/mL). Spectral displacements with the increase of washing cycles are marked with arrows.

In order to investigate the effect of the GNPs on the secondary structure of the proteins, we performed CD spectroscopy analysis. The protein concentrations were more dilute than usually used in CD analysis (typically 0.1 mg/mL 1 mm pathlength); however, as can be seen in Figure 8, there are two peaks at 208 and 222 nm on the BSA spectrum, which are identified as characteristic peaks of the α-helical conformation in the protein, caused by the π–π* and n–π* transitions, respectively, of peptide chains [60]. The 193 nm alpha helix π–π* peak is also apparent. After the first wash, the protein with both GNPs was mostly in its original conformational state, though protein was removed from the sample. With the increasing washing steps, the AuNP spectra lost further intensity with the 208 nm peak moving to lower wavelength, and the 193 nm intensity reduced in accord with the protein unfolding. Thus we conclude that as the soft corona was removed, the proteins in the hard corona dominated with a slight change in their secondary structure. By way of contrast, the protein with AuNS lost CD intensity (consistent with it being washed away), but what remained was still folded—presumably less tightly bound to the AuNS or free in the solution.

**Figure 9:**
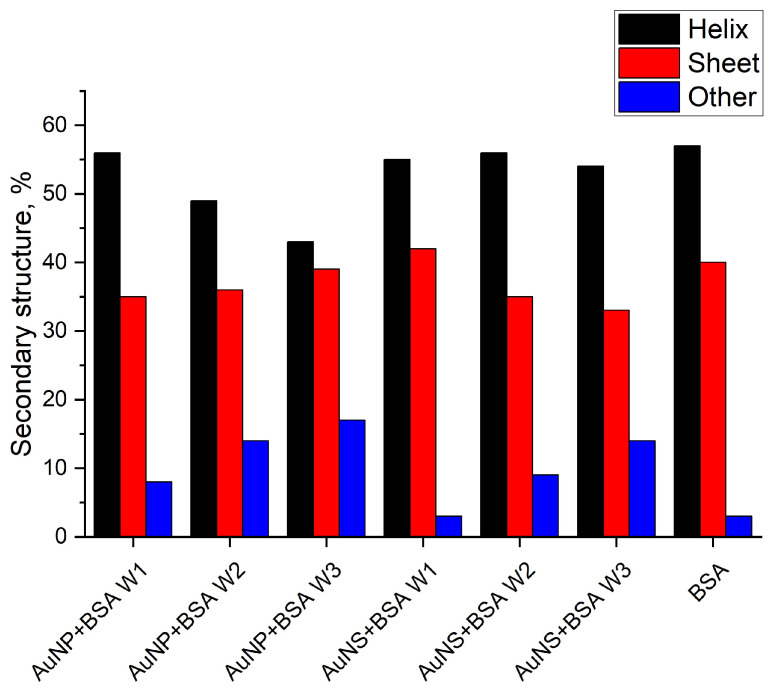
Secondary structure composition of BSA on the GNPs surface estimated via SOMspec algorithm (for details see ESI: Appendix E).

We have used the SOMspec algorithm to determine the secondary structure from CD spectra summarized in Figure 9 (fitting graphs and additional information are given in the ESI Appendix E). The fitting data result supports unfolding of the protein absorbed on the surface of the AuNP. With the increase in washing steps, loosely bound protein, that was not much affected by interaction with GNPs, was washed off. The protein that was tightly bound to the surface of GNPs lost its original secondary structure.

### 3.3. GNP Interaction with Small Molecules after Incubation with Protein

**Figure 10:**
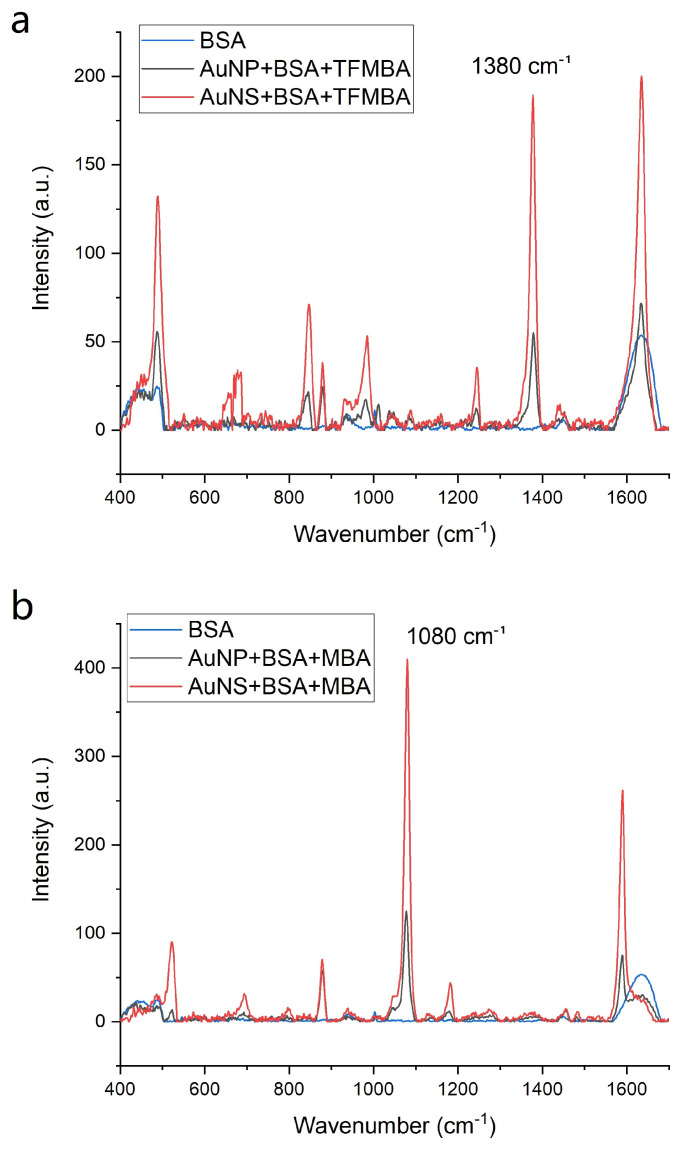
Raman spectra of (**a**) TFMBA (1 mM) and (**b**) MBA (1 mM) with BSA (4.5 mg/mL) incubated AuNP (black line) and AuNS (red line); the BSA spectrum (4.5 mg/mL, blue) is overlaid.

Since the AuNS did not have significant PC near their SERS “hot spots”, we explored whether this particle geometry prevented BSA from fouling the GNPs. AuNP and AuNS were sequentially incubated with BSA and small molecule reagents TFMBA or MBA. As can be seen in Figure 10, the Raman signal intensity of the small molecule characteristic peaks was much larger, when incubated with AuNS samples. After incubation with the particles, we washed the GNP/protein/analyte sample as described in the “Materials and Methods” section and resuspended the mixture in water, which removed ethanol from the sample that was tested, leaving only analytes, that were bound to the surface of the GNPs (black and red lines). Considering concentration differences, the AuNS signal enhancement after incubation with protein was at least 10-fold larger than that of AuNP (Table 4). Although both AuNS and AuNP showed decreases in their EFs after incubation with the protein, these decreases were significantly lower in AuNS samples. Thus, we conclude that AuNS have the potential to be used effectively with SERS sensing/detecting/imaging systems for detection of small molecules in biological media, as the large protein molecules did not completely shield the “hot spots”.

## 4. Conclusions

Gold nanoparticles have the potential to be used in many bio-applications such as sensing, imaging and detecting. Advanced properties of anisotropic nanoparticles, in particular, nanostars, increase the probability of achieving those goals.

In this study, we have demonstrated reproducible syntheses of spheroidal and star-shaped GNPs with consistent size and morphology parameters. The particles enhanced Raman signals by about a factor of $10^{4}$. The exciting result of this work was the discovery of reduced fouling of gold nanostars relative to spheroidal particles. After incubation with protein, AuNP had layers of proteins around them, fully or partly covering the surfaces of the nanoparticles (verified with SERS and cryo-TEM microscopy); AuNS did not. Due to this characteristic, the protein had less of an effect on the AuNS SERS enhancement of small molecules. After interaction with BSA, the Raman signal enhancement of the star-shaped particles was about 10-fold larger than that of the spheroidal particles. We speculate that the particles’ shape might be the key to their ability to limit protein corona formation, as parameters such as capping agent and incubation conditions were similar for both particle types. It should be noted that the proteins that bound closely to the AuNP surface underwent slight conformational changes with an element of unfolding which might have affected cellular uptake and the cytotoxicity of GNPs. In summary, this work provides more understanding about protein corona formation that might occur in the biological media on GNPs of different shapes and can help in the design nanoparticles that limit protein fouling for future studies of plasmonic particles in protein-rich media.

## Figures and Tables

**Table 1:** Size and surface charge results. DLS values are the averages of 10 measurements per sample; the errors are single standard deviations of these measurements. The TEM particle sizes are averages of between 20 and 100 particles per sample; the errors are single standard deviations.

Sample	Size, nm		Surface Charge (ZP), mV
	**DLS**	**TEM**	
AuNP	94 ± 5 (PDI: 0.254)	93 ± 17	−30.6 ± 1.9
AuNS	139 ± 27 (PDI: 0.396)	92 ± 21, core size ∼ 50 nm	−30.6 ± 0.7

**Table 2:** SERS enhancement factors (EF) of GNPs calculated from magnitudes of the signal intensities of the TFMBA characteristic peak at 1380 ${\mathrm{cm}}^{-1}$ and MBA at 1080 ${\mathrm{cm}}^{-1}$ and scaled in accordance to GNP concentrations. See ESI Appendix A.2 for more details.

Sample	EF
AuNP + TFMBA	$4.4\times 10^{4}$
AuNS+TFMBA	$6.5\times 10^{4}$
AuNP + MBA	$8.2\times 10^{4}$
AuNS+MBA	$1.2\times 10^{5}$

**Table 3:** SERS characteristic peaks of BSA observed in Figure 7.

Wavelength, ${\mathrm{cm}}^{-1}$	Assignment [57,58,59]
490	ν(S–S)
506	ν(S–S)
602	δ(COO–)
753	Trp, ν(C–S)
941	δ(C–C–N) symm, α-helical skeletal
996	R breathing
1003	Phe: indole asymm ring
1183	Tyr, ν(–C–N)
1300–1200	Trp, Phe: δ(R), Amide III—region
1309	Wag (CH_2_)
1360–1340	Trp, doublet
1383	δ(CH_3_), ν(COO–)
1437	asymm δ(CH_3_), bend (CH_2_)
1560	Trp: ν(R), ν(r), amide II

**Table 4:** SERS enhancement factor (EF) of protein-coated GNPs calculated through signal intensity of characteristic peaks: 1380 ${\mathrm{cm}}^{-1}$ for TFMBA and 1080 ${\mathrm{cm}}^{-1}$ for MBA.

Sample	EF	EF Ratios	
		**Comparison between** **Protein** **Incubated and bare GNP**	**Comparison** **between** **AuNS and AuNP**
AuNP+TFMBA	4.4 × 10^4^	44-fold decrease	10-fold larger
AuNP+BSA+TFMBA	1.0 × 10^3^		
AuNS+TFMBA	6.5 × 10^4^	7-fold decrease	
AuNS+BSA+TFMBA	9.5 × 10^3^		
AuNP+MBA	8.2 × 10^4^	39-fold decrease	12-fold larger
AuNP+BSA+MBA	2.1 × 10^3^		
AuNS+MBA	1.2 × 10^5^	6-fold decrease	
AuNS+BSA+MBA	2.6 × 10^4^		

## Data Availability

Data are available from the authors upon reasonable request.

## Supplementary Materials

The following are available online at https://www.mdpi.com/article/10.3390/nano11102565/s1, Figure S1: inite track length adjustment (FTLA) Concentration/Size graphs for NTA analysis of (a) AuNP and (b) AuNS, Table S1: GNP concentrations obtained from NTA analysis, Figure S2: Gold nanostar model for the surface area estimation, Figure S3: Raman spectra of solid TFMBA and MBA powder, Table S2: DLS size-destribution graphs: AuNP and AuNS batches prepared at different times. AuNPs’ diameter is around 94 nm and AuNSs’ diameter is 139 nm with the error between batched 5 nm and 27 nm respectively, Figure S4: TEM-based histogram of AuNP size distribution, Figure S5: TEM of gold nanostars prepared using (a) vortex, (b) constant stirring, (c) stirring with stops type A, (d) stirring with stops type B, (e) stirring with stops type C, Table S3: UV-Vis absorbance spectra of protein-coated particles, Figure S6: Cryo-TEM imagies of GNP incubated with BSA: (a) AuNP+BSA W1, (b) AuNP+BSA W2, (c) AuNP+BSA W3, (d) AuNS+BSA W1, (e) AuNS+BSA W2, (f) AuNS+BSA W3. Protein coated particles after first (W1), second (W2) and third (W3) wash, Table S4: Secondary structure composition of BSA on the GNPs surface estimated via SOMspec algorithm.

## Abbreviations

The following abbreviations are used in this manuscript:

AuNP	Spheroidal Gold Nanoparticles
AuNS	Star-shaped Gold Nanoparticle
BSA	Bovine Serum Albumin
CCK-8	Cell Counting Kit-8
DLS	Dynamic Light Scattering
DMSO	Dimethyl Sulfoxide
EF	Enhancement Factor
GNPs	Gold Nanoparticles
LSPR	Localized Surface Plasmon Resonance
MBA	4-Mercaptobenzoic Acid
NIR	Near Infrared
NP	Nanoparticle
NTA	Nanoparticles Tracking Analysis
PBS	Phosphate-Buffered Saline
PC	Protein Corona
PDI	Polydispersity Index
SERS	Serface-Enhanced Raman Spectroscopy
SI	Support Information
SOM	Self-Organizing Map
TEM	Transmission Electron Microscopy
TFMBA	2,3,5,6-Tetrafluoro-4-Mercaptobenzoic Acid
UV–Vis	Ultraviolet–Visible Absorption Spectroscopy
ZP	Zeta Potential
