# Supplementary material for: Gold Nanostars with Reduced Fouling Facilitate Small Molecule Detection in the Presence of Protein
Source: Nanomaterials (Basel). 2021 Sep 29;11(10):2565. doi: 10.3390/nano11102565 (PMC8538065; doi:10.3390/nano11102565)
Supplement: Supplementary file 1 [file nanomaterials-11-02565-s001.zip › nanomaterials-1369847-supplementary.pdf]

Supplementary Materials

Gold Nanostars with Reduced Fouling Facilitate Small Molecule Detection in the Presence of Protein

Anastasiia Tukova [1,*], Inga Christine Kuschnerus [2,3], Alfonso Garcia-Bennett [1] and Yuling Wang [1,*] and Alison Rodger [1]

1 Department of Molecular Sciences, Faculty of Science and Engineering, Macquarie University, Sydney, NSW 2019, Australia; alf.garcia@mq.edu.au (A.G.-B.); alison.rodger@mq.edu.au (A.R.)
2 Electron Microscopy Unit, University of New South Wales, Sydney, NSW 2052, Australia; i.kuschnerus@unsw.edu.au
3 School of Materials Science and Engineering, University of New South Wales, Sydney, NSW 2052, Australia
* Correspondence: Correspondence: anastasiia.tukova@hdr.mq.edu.au (A.T.); yuling.wang@mq.edu.au (Y.W.)

Contents

Non-fouling gold nanostars facilitate small molecules detection in biological media 1
Appendix A - Calculations 2
Appendix A.1 NTA mesaurments 2
Appendix A.2 Enhancement factor calculation 3
Appendix A.3 TFMBA and MBA solid powder Raman spectra 5
Appendix B – Synthesis of GNPs 6
Appendix B.1 Reproducibility 6
Appendix B.2 Synthesis Of Gold Nanostars (Effect of Mixing) 7
Appendix C – UV-visible Absorbance Spectra of GNPs after Protein Coating 9
Appendix D - Cryo-TEM images of Protein Coated GNPs 10
Appendix E – SOMspec fitting data 11
References 13

Appendix A - Calculations

Appendix A.1 NTA mesaurments

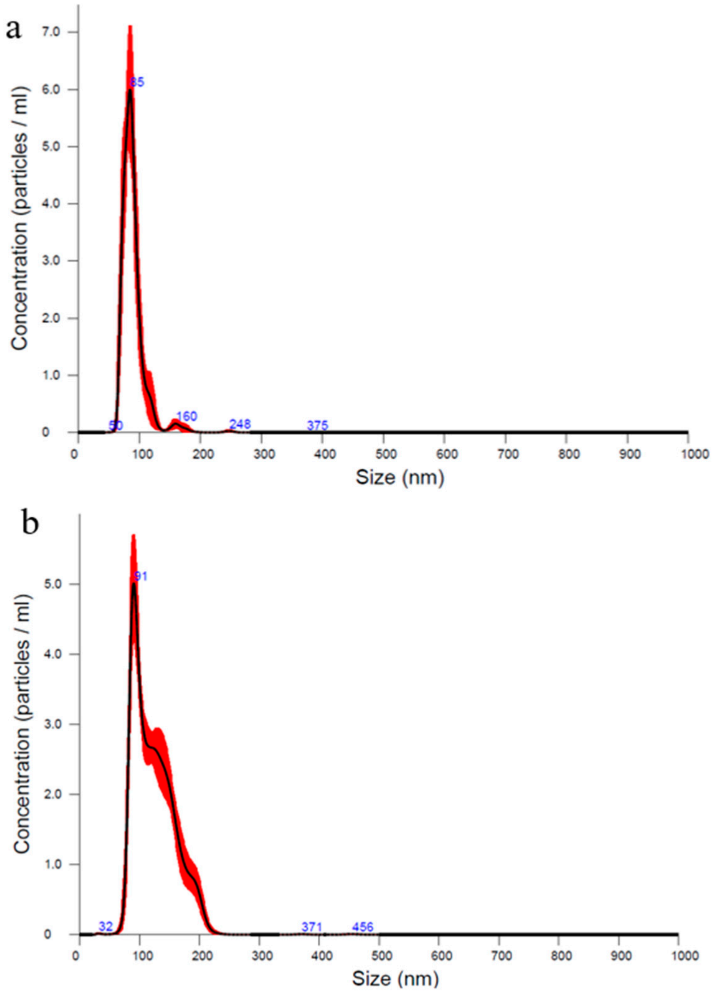

Figure S1. Finite track length adjustment (FTLA) Concentration/Size graphs for NTA analysis of a) AuNP and b) AuNS.

Table S1. GNP concentrations obtained from NTA analysis.

Samples	NTA, particles/mL
AuNP	1.75e+10 +/- 1.08e+08
AuNS	2.89e+09 +/- 8.52e+07

Appendix A.2 Enhancement factor calculation

The SERS enhancement factor was determined from the ratio of the TFMBA SERS signal and Raman signal for AuNPs and AuNSs at 1380 cm^{-1}.

I_{SERS} – SERS intensity of TFMBA at 1380 cm^{-1} – read from the graph,

I_{Raman} – Raman intensity of TFMBA at 1380 cm^{-1} – read from the graph,

The diameter (d) of the laser spot in the liquid is 40 μm, pathlength (h) is 10 mm,

$$V_{\text{Laser spot}} = \pi(\frac{d}{2})^2 \cdot h = \pi(20\ \mu m)^2 \cdot 10\ mm = 1.26 \times 10^{-5}\ cm^3,$$

$$Mole\ of\ TFMBA\ in\ laser\ spot = C_{TFMBA} \cdot V_{Laser\ spot} = 0.001 \frac{mol}{L} \cdot 1.26 \cdot 10^{-5} cm^3 = 1.26 \times 10^{-11}\ mol,$$

$$N_{Raman} = Mole\ of\ TFMBA\ in\ laser\ spot \cdot Avagadro's\ constan = 1.26 \times 10^{-11} \cdot 6.02 \times 10^{23} = 8.69 \times 10^{12}\ molecules,$$

$$S_{spherical\ nanoparticle} = 4\pi r^2 = 4\pi(43.5nm)^2 = 20096\ nm^2 - surface\ area\ of\ spherical\ particle,$$

where r – particle radius from TEM data,

$$N_{TFMBA} = \frac{S_{nanoparticle}}{S_{TFMBA}} = \frac{27158\ nm^2}{0.383\ nm^2} = 70908\ molecules,$$

N_{SERS} and N_{Raman} are the amount of Raman reporter molecules during SERS measurements (with GNPs), that were excited in laser spot and number of reporter molecules during Raman measurements (without GNPs), respectively.

$$C_{TFMBA\ after\ wash} = \frac{N_{TFMBA} \cdot C_{nanoparticle}}{N_A - Avogadro's\ number} = 1.027\ \mu M$$ – concentration of TFMBA bound to AuNP

$$N_{nanoparticle\ in\ laser\ spot} = C_{nanoparticle} \cdot V_{laser\ spot} = 8.72 \times 10^9 \frac{nanopartile}{cm^3} \cdot 1.26 \times 10^{-5} cm^3 = 1.10 \times 10^5\ nanoparticles,$$

$$N_{SERS} = N_{nanoparticle\ in\ laser\ spot} \cdot N_{TFMBA} = 1.10 \times 10^5\ nanoparticles \cdot 70908\ molecules = 7.77 \times 10^9\ molecules,$$

$$EF\ (AuNP) = \frac{I_{SERS}}{I_{Raman}} \cdot \frac{N_{Raman}}{N_{SERS}} = \frac{2347}{30} \cdot \frac{8.69 \times 10^{12}\ molecules}{7.77 \times 10^9\ molecules} = 8.76 \times 10^4.$$

$$S_{star\ nanoparticle} = 4\pi a^2 - \sum_{i=1}^{n}\left\{2\pi a(a - \sqrt{a^2 - R_i^2})\right\} + \sum_{i}^{n}\left\{\pi(r_i + R_i)\sqrt{(R_i - r_i)^2 + h_i^2} + 2\pi r_i^2\right\} = 60620\ nm^2 - - particles\ surface\ area\ of\ star\ shaped\ particle,$$

where a is the radius of spherical core, R is the base radius of a spike, r is the radius of the hemispherical tip of a spike, and h is the distance between spike's base and its hemispherical tip's base (Figure S2).[1]

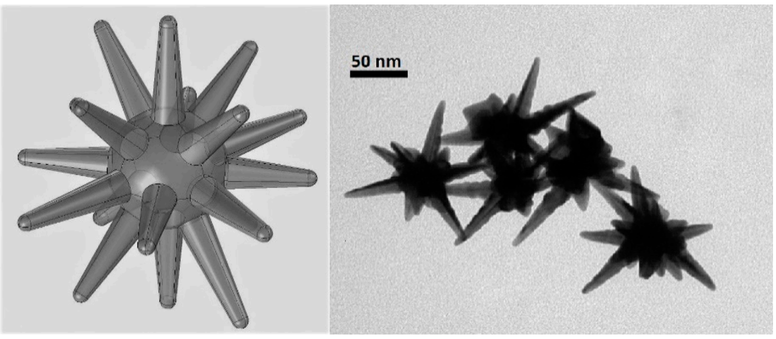

Figure S2. Gold nanostar model for the surface area estimation.

$$N_{TFMBA} = \frac{S_{nanoparticle}}{S_{TFMBA}} = \frac{60620\ nm^2}{0.383\ nm^2} = 158275\ molecules,$$

N_{SERS} and N_{Raman} are the amount of Raman reporter molecules during SERS measurements (with GNPs), that were excited in laser spot and number of reporter molecules during Raman measurements (without GNPs), respectively.

$$C_{TFMBA\ after\ wash} = \frac{N_{TFMBA} \cdot C_{nanoparticle}}{N_A - Avogadro's\ number} = 0.98\ \mu M$$ – concentration of TFMBA bound to AuNS

$$N_{nanoparticle\ in\ laser\ spot} = C_{nanoparticle} \cdot V_{laser\ spot} = 1.32 \times 10^9 \frac{nanopartile}{cm^3} \cdot 1.26 \times 10^{-5} cm^3 = 1.65 \times 10^4\ nanoparticles,$$

$$N_{SERS} = N_{nanoparticle\ in\ laser\ spot} \cdot N_{TFMBA} = 1.65 \times 10^4\ nanoparticles \cdot 158275\ molecules = 2.62 \times 10^9\ molecules,$$

$$EF\ (AuNS) = \frac{I_{SERS}}{I_{Raman}} \cdot \frac{N_{Raman}}{N_{SERS}} = \frac{1294}{30} \cdot \frac{8.69 \times 10^{12}\ molecules}{2.62 \times 10^9\ molecules} = 1.43 \times 10^5.$$

Appendix A.3 TFMBA and MBA solid powder Raman spectra

Raman spectra were collected with a portable IM-52 Raman microscope (Snowy Range Instruments, USA), 70 mW of 785 nm incident laser power with an integration time of 20 msec.

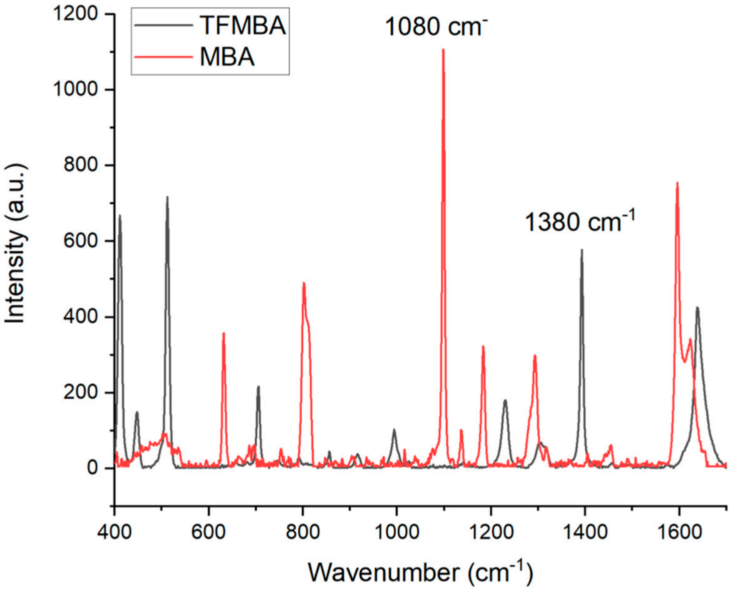

Figure S3. Raman spectra of solid TFMBA and MBA powder.

Appendix B – Synthesis of GNPs

Appendix B.1 Reproducibility

The reproducibility of the syntheses of AuNP (spheroids) and AuNS (stars) particles has been demonstrated through dynamic light scattering (DLS) results. The four batches of AuNP and AuNS that have been used in the experiments had similar particle-size distributions from batch-to-batch (Table S2).

Table S2. DLS size-destribution graphs: AuNP and AuNS batches prepared at different times. AuNPs' diameter is around 94 nm and AuNSs' diameter is 139 nm with the error between batched 5 nm and 27 nm respectively.

	**AuNP (spheroids)**	**AuNS (stars)**
Batch 1	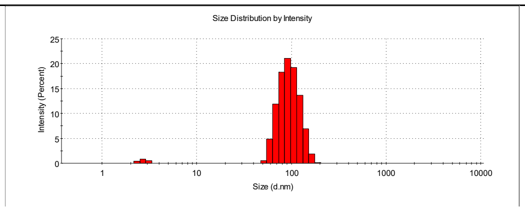<br>Size: 96.5 nm	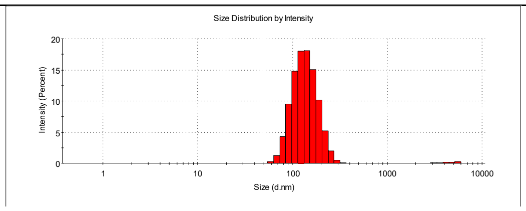<br>Size: 123.1nm

Batch 2	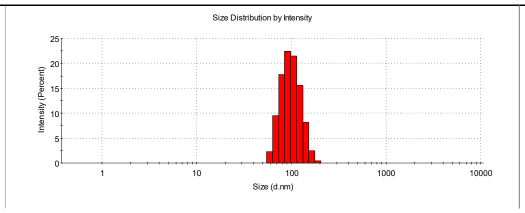 Size: 100.4 nm	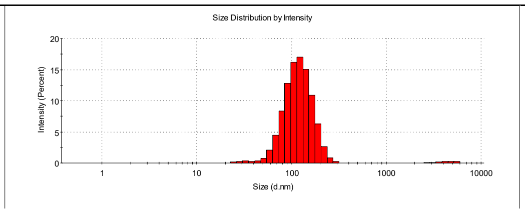 Size: 123.1 nm
Batch 3	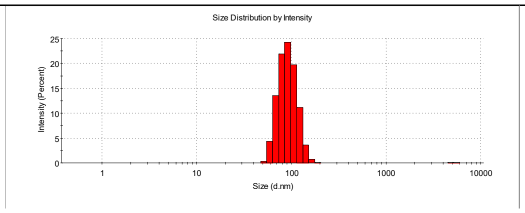 Size: 92.7 nm	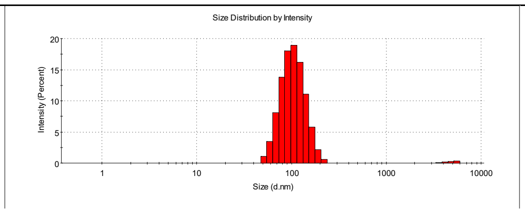 Size: 109.3 nm
Batch 4	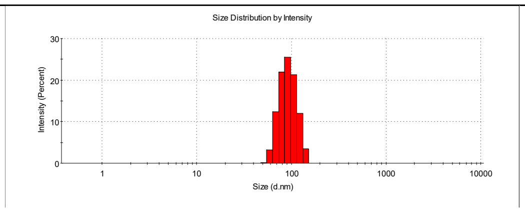 Size: 87.8 nm	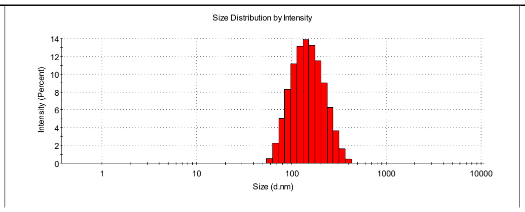 Size: 182.2 nm

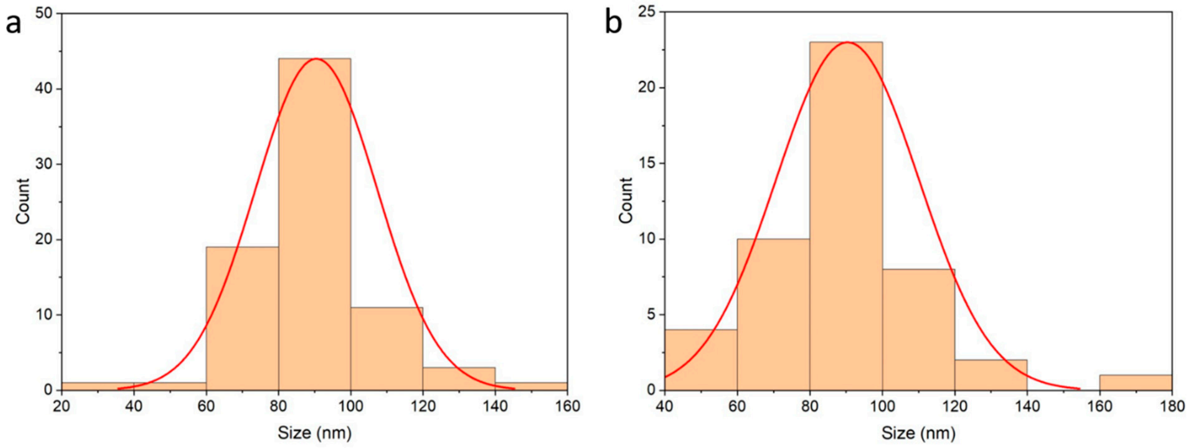

Figure S4. TEM-based histogram of AuNP size distribution.

Appendix B.2 Synthesis Of Gold Nanostars (Effect of Mixing)

We varied the way of reagent-mixing whilst keeping the same chemical ratio, injection time and order of reagent addition, to understand how mixing might influence the formation of branched or other structures.

a) Vortexing

The reagents were mixed by introducing each ingredient and vortexing for 30 s, as described in the Materials and Methods section of the main paper. Figure S5a shows NPs with multiple edges a core of approximately 60 nm and a large variety of total particles sizes. The preparation of particles by vortexing is difficult to perform reproducibly due to the need to hold the tube with reaction mixture throughout the preparation and due to person-to-person variability.

b) Constant stirring

The reaction mixture was constantly stirred with magnetic stir bar at a speed of 1000 rpm and the components were introduced in turn every 30 s. Stirring was continued for 30 s after the final addition. The resulting TEM image (Figure S5b) shows the formation of two types of structures: coral-shaped large round clusters and small spherical beads. The constant stirring seems to prevent proper particle growth, resulting in the formation of small beads and their aggregation into large clusters.

c) Stirring with stops to add reagents (A)

The stirring (30 s at 1000 rpm) was stopped 3 times for a few seconds to add each reagent. The mixing was for 30 s after each reagent injection. In this case the particles were formed with long sharp spikes, which is the target morphology (due to surface plasmon properties of that shape)[2] (Figure S5c).

d) Stirring with one stop to add the shaping agent (B)

The stirring (30 s at 1000 rpm) was stopped only to add shaping agent to the mixture ($AgNO_3$), whereas citric acid ($C_6H_8O_6$) was injected during stirring . The mixture was stirred for 30 s after each addition was complete. The structures formed are heterogeneous: some particles formed as nanostars, while some formed as irregularly shaped anisotropic particles with angled surfaces. The polydispersity is high (Figure S5d).

e) Stirring with one stop to add reducing agent (C)

Stirring (30s at 1000 rpm) was stopped to add citric acid while the mixture was constantly stirred when adding the first two reagents in the order $HAuCl_4$ and $AgNO_3$. The mixture was stirred for 30 s after each reagent addition. The particles look like crumpled or “folded” paper. The polydispersity is very high (Figure S5e).

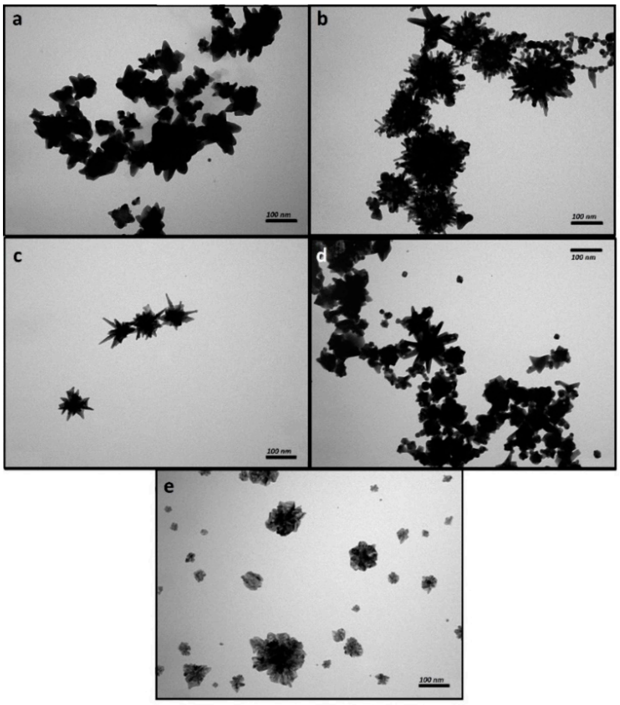

Figure S5. TEM of gold nanostars prepared using a) vortex, b) constant stirring, c) stirring with stops type A, d) stirring with stops type B, e) stirring with stops type C.

Preparing nanostructures with a magnetic stirrer rather than by vortexing is easier to perform and allows better control of mixing speed and reagent-injection. The optimal morphology was concluded to be achieved by stirring with stops after each reagent injection (stirring method (C)). Visually the particles are monodispersed and have similar shape. The branches are long and sharp, which is the targeted morphology. The size of the anisotropic particles prepared by this method is also close to the size of spherical particles which facilitates comparison of particle performance.

Appendix C – UV-visible Absorbance Spectra of GNPs after Protein Coating

The gold nanoparticles were incubated with protein and washed one, two or three times to remove soft protein corona. The UV-Vis absorbance spectra of the washed samples are demonstrated in Table S3.

Table S3. UV-Vis absorbance spectra of protein-coated particles.

	**AuNP+BSA**	**AuNS+BSA**
Wash1	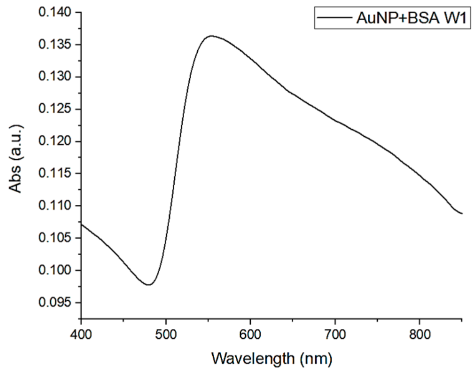	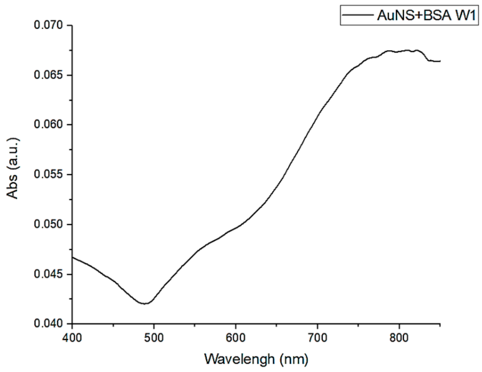
Wash2	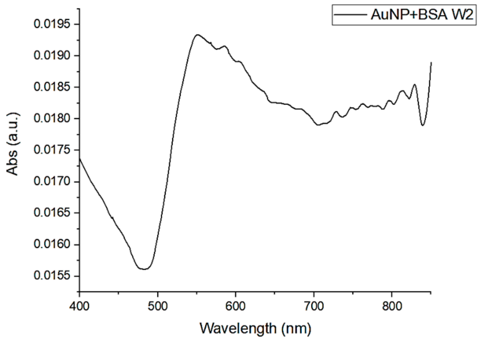	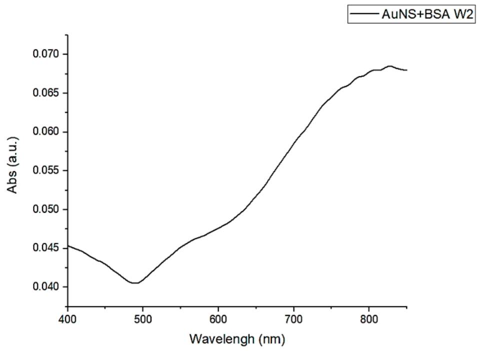
Wash3	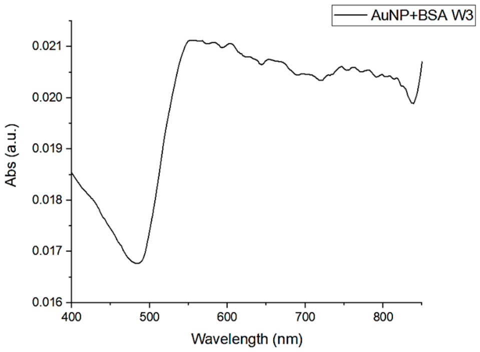	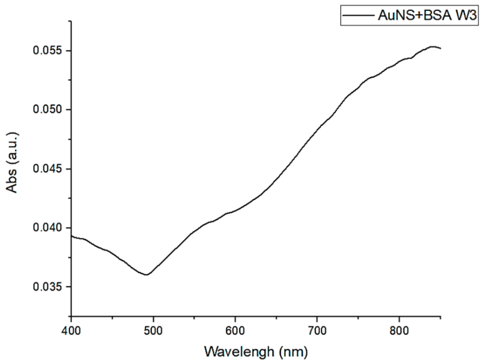

Appendix D - Cryo-TEM images of Protein Coated GNPs

Additional cryo-TEM images of AuNP and AuNS incubated with BSA and washed one, two and three times are shown in Figure S6. No protein corona can be seen on the surface or near the nanostars.

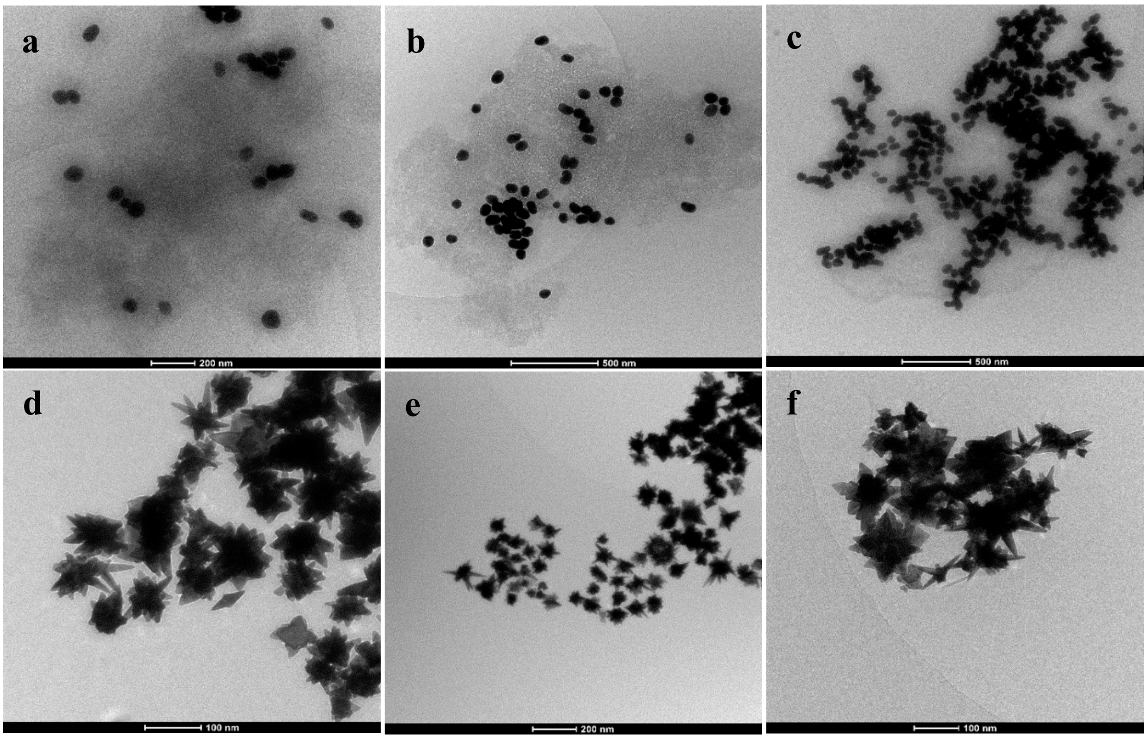

Figure S6. Cryo-TEM imagies of GNP incubated with BSA: a)AuNP+BSA W1, b) AuNP+BSA W2, c) AuNP+BSA W3, d) AuNS+BSA W1, e) AuNS+BSA W2, f) AuNS+BSA W3. Protein coated particles after first (W1), second (W2) and third (W3) wash.

Appendix E – SOMspec fitting data

The secondary structure was analyzed via a self-organising map (SOM) structure-fitting methodology (SOMSpec) (Table S4).[3] Each sample CD spectra was analysed over 53-protein reference set in the wavelength range from 240 nm to 190 nm with 1 nm step.

Table S4. Secondary structure composition of BSA on the GNPs surface estimated via SOMspec algorithm.

	Graph	Protein concentration mg/mL	Helix, %	Sheet, %	Other, %
AuNP+ BSA W1	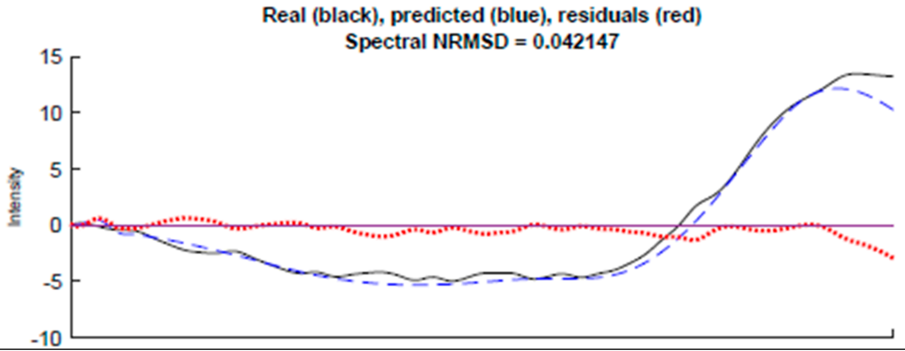	0.01	56	35	8
AuNP+ BSA W2	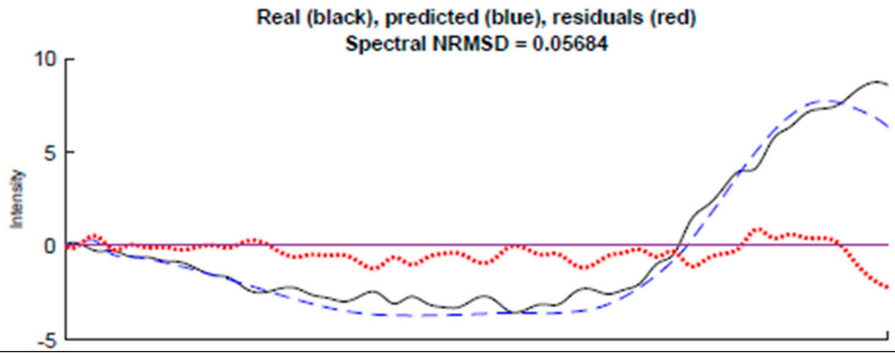	0.008	49	36	14
AuNP+ BSA W3	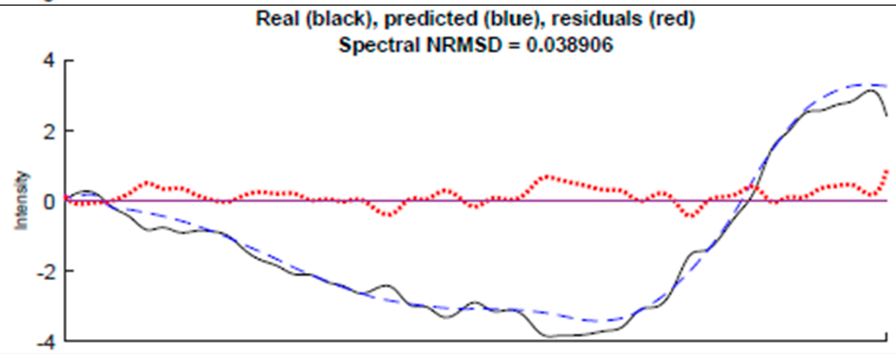	0.014	43	39	17
AuNS+ BSA W1	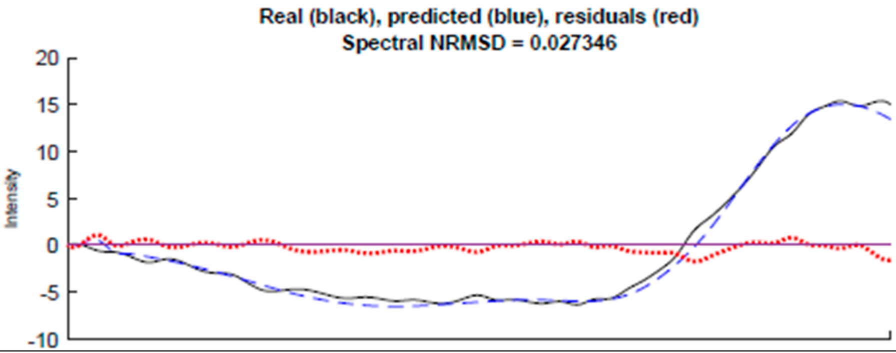	0.008	55	42	3

AuNS+ BSA W2	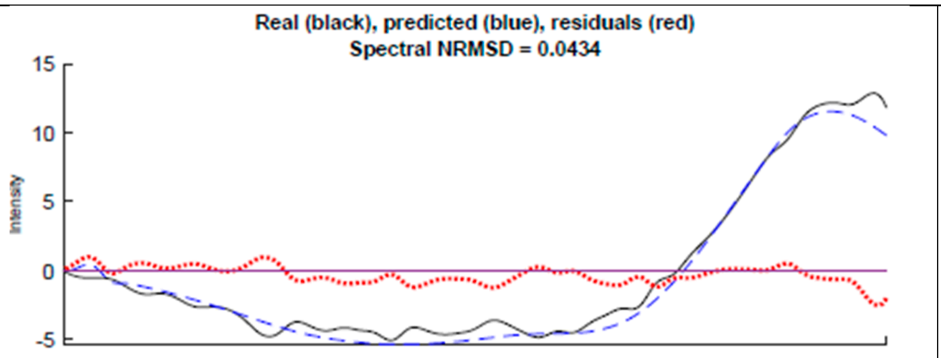	0.006	56	35	9
AuNS+ BSA W3	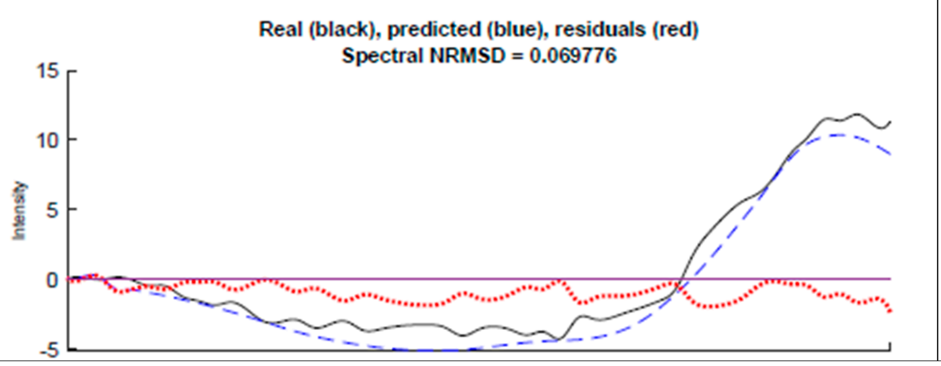	0.006	54	33	14
BSA	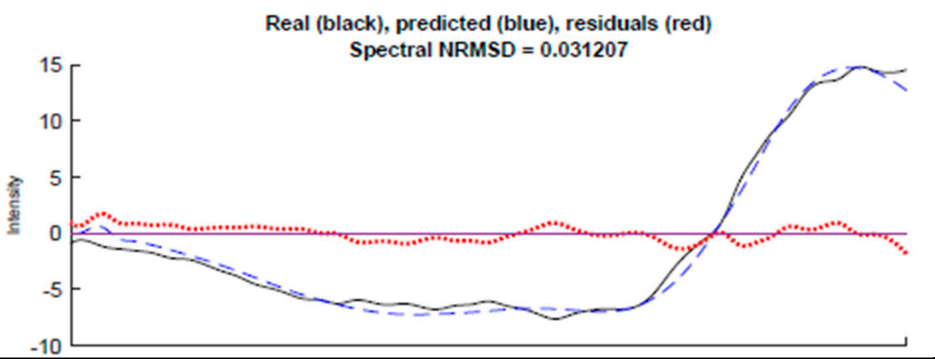	0.005	57	40	3

References

1. Tsoulos T V, Han L, Weir J, Xin HL, Fabris L. A closer look at the physical and optical properties of gold nanostars: an experimental and computational study. *Nanoscale*. 2017;9(11):3766-3773. doi:10.1039/C6NR09091E
2. Guerrero-Martínez A, Barbosa S, Pastoriza-Santos I, Liz-Marzán LM. Nanostars shine bright for you: Colloidal synthesis, properties and applications of branched metallic nanoparticles. *Curr Opin Colloid Interface Sci*. 2011;16(2):118-127. doi:https://doi.org/10.1016/j.cocis.2010.12.007
3. Hall V, Sklepari M, Rodger A. Protein secondary structure prediction from circular dichroism spectra using a self-organizing map with concentration correction. *Chirality*. 2014;26(9):471-482. doi:10.1002/chir.22338
